# Metabolic Profiling Combined With Machine Learning for Assessing Clonal Hematopoiesis Status in Chronic Immune Thrombocytopenia: An Exploratory Analysis

**DOI:** 10.1002/mco2.71129

**Published:** 2026-11-08

**Authors:** Jia Chen, Qing Wen, Ting Sun, Xiaofan Liu, Feng Xue, Wei Liu, Rongfeng Fu, Mankai Ju, Xinyue Dai, Ying Chi, Wentian Wang, Renchi Yang, Yunfei Chen, Lei Zhang

**Affiliations:** ^1^ State Key Laboratory of Experimental Hematology, National Clinical Research Center for Blood Diseases, Haihe Laboratory of Cell Ecosystem, Institute of Hematology & Blood Diseases Hospital, Tianjin Key Laboratory of Gene Therapy for Blood Diseases, CAMS Key Laboratory of Gene Therapy for Blood Diseases Chinese Academy of Medical Sciences & Peking Union Medical College Tianjin China; ^2^ Tianjin Institutes of Health Science Tianjin China; ^3^ School of Population Medicine and Public Health Chinese Academy of Medical Sciences and Peking Union Medical College Beijing China

**Keywords:** clonal hematopoiesis, DNMT3A mutation, immune thrombocytopenia, machine learning, metabolomics, multi‐model analysis

## Abstract

Clonal hematopoiesis (CH) has emerged as a potential contributor to the pathogenesis of immune thrombocytopenia (ITP), yet the clinical and metabolic features of ITP patients harboring CH‐associated mutations remain poorly characterized. We retrospectively analyzed a genomic discovery cohort of 1817 patients with chronic ITP, identifying CH in 196 individuals (10.8%), with DNMT3A as the most frequently mutated gene. In a metabolomic profiling cohort of 539 patients (492 ITP‐non‐CH, 47 ITP‐CH), we characterized plasma metabolic signatures using untargeted LC‐MS/MS and systematically evaluated the discriminative capacity of integrated metabolic‐clinical features through multiple machine learning algorithms. Differential analysis identified 14 plasma metabolites between ITP‐non‐CH and ITP‐CH patients. The cohort was randomly partitioned into a training set (*n* = 377) and an independent test set (*n* = 162). Among eight evaluated algorithms, logistic regression with balanced class weighting and 14 metabolites + 3 clinical features (sex, age, PLT) achieved the optimal discriminative balance, with a test AUC of 0.721 (95% CI: 0.57–0.87). Our study reveals distinct hematologic and metabolic dysregulation between ITP‐CH and ITP‐non‐CH, and highlights the potential of integrative hematologic‐metabolic signatures for improving disease stratification in ITP.

## Graphical abstract

Clonal hematopoiesis (CH) may contribute to immune thrombocytopenia (ITP). Among 1817 patients with chronic ITP, CH occurred in 10.8%. Plasma metabolomes of 539 patients were profiled by untargeted LC‐MS/MS, revealing 14 metabolites distinguishing CH from non‐CH. Logistic regression combining these metabolites with sex, age, and platelet count achieved a test AUC of 0.721, suggesting metabolic‐clinical signatures may improve ITP stratification.

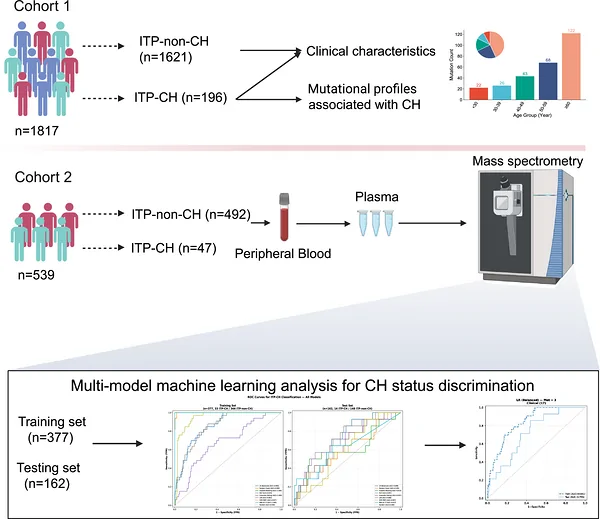

## 1 Introduction

Clonal hematopoiesis (CH) refers to the presence of somatic mutations in hematopoietic stem and progenitor cells (HSPCs) that confer a proliferative advantage and drive their clonal expansion [1]. With the widespread adoption of high‐throughput sequencing, CH has been detected with increasing frequency across diverse clinical settings. It has been associated with inflammation and systemic inflammatory diseases [2], as well as with evolution to hematologic malignancies such as acute myeloid leukemia (AML), myelodysplastic syndrome (MDS), or myeloproliferative neoplasm (MPN) [3, 4]. Notably, CH has also been reported in association with a range of autoimmune diseases [5].

Immune thrombocytopenia (ITP) is an autoimmune disease characterized by decreased platelet counts and an increased risk of bleeding [6]. Several recent studies have examined the prevalence and clinical significance of CH in ITP. A multicentric retrospective observational study of 167 adult patients found that 18.5% carried somatic mutations typical of CH, and that these patients had a higher thrombotic risk and a more refractory or relapsing disease course [7]. Wang et al. reported a CH prevalence of 10.78% in ITP and showed that CH was associated with poorer responses to therapy, inferior overall survival, and progression to MDS or AML [8]. Together, these findings suggest that ITP with CH may represent a distinct subtype that differs from typical ITP in its biological features, treatment response, and prognosis. Understanding clonal hematopoiesis in the context of ITP therefore not only advances our knowledge of disease pathogenesis, but may also inform personalized treatment and prognostic management. However, because next‐generation sequencing (NGS) is not readily accessible in many clinical settings, identifying ITP patients with clonal hematopoiesis remains clinically challenging and is often overlooked.

Clonal hematopoiesis and cellular metabolism are closely intertwined. The genes most commonly mutated in CH, including DNMT3A, TET2, and spliceosome components, regulate processes that are themselves coupled to metabolic state, and CH has increasingly been linked to metabolic reprogramming in hematopoietic stem cells, particularly an enhanced reliance on oxidative phosphorylation [9, 10]. Because such metabolic alterations can be reflected in the circulating metabolome, plasma metabolites may offer a non‐invasive window into the presence and functional consequences of clonal hematopoiesis.

Plasma metabolomics, the systematic analysis of small‐molecule metabolites in blood, has emerged as a powerful tool for investigating disease. Metabolomic profiling has provided insights into the biochemical changes associated with ITP. One study used gas chromatography‐mass spectrometry (GC‐MS) to profile plasma from 98 ITP patients and 30 healthy controls, identifying 17 differential metabolites involved in lipid, amino acid, and energy metabolism [11]. In chronic ITP, six metabolites (lactic acid, glyceric acid, L‐valine, pyruvic acid, uric acid, and α‐ketoglutaric acid) predicted glucocorticoid response, with AUCs ranging from 0.799 to 0.868 [11]. Our earlier work demonstrated that metabolic perturbations in bone marrow supernatants correlate with ITP progression and hold promise as predictive biomarkers of therapeutic response [12]. Nevertheless, the relationship between CH status and metabolism in ITP has not been previously explored.

In this study, we characterized the clinical and genetic profiles of ITP with and without clonal hematopoiesis in a large clinical cohort. By integrating plasma metabolomic analysis with multi‐model machine learning, we systematically evaluated whether integrated metabolic‐clinical signatures could distinguish ITP‐non‐CH from ITP‐CH patients. Metabolomic signals that reflect clonal and immune activity may provide information complementary to conventional clinical phenotyping, although NGS remains the gold standard for confirming CH.

## 2 Results

### 2.1 Patient Characteristics and Mutational Profiles in Cohort 1

A total of 1817 patients with chronic ITP (cohort 1) were enrolled before 2021 (Figure 1; Table S1). 196 (10.8%) patients had CH (ITP‐CH). The median age at diagnosis was 44 years (IQR: 28–56 years) in ITP patients without CH (ITP‐non‐CH), and 57 years (IQR: 45.3‐66.8 years) in ITP‐CH. A comprehensive summary of clinical features and available treatments are provided in Table S1. Figure 2A displays the mutation profiles of CH‐related genes in ITP patients, with *DNMT3A* mutations being the most frequent (29%), followed by *TET2* (15%), *ASXL1* (8%), *BCOR* (7%), and *U2AF1* (6%). The number of gene mutations increased with age (Figure 2B). The mutation burden and frequency of epigenetic regulators: *DNMT3A*, *TET2*, and *ASXL1*, exhibited a significant age‐dependent increase (Figure 2C–E). In contrast, no such trend was observed for *BCOR* or *U2AF1* mutations (Figure 2F,G). Missense mutations represent the most prevalent alteration type in *DNMT3A*, with the R882H substitution being the most frequently observed variant, followed by R882C, R736C, and R736H (Figure S1, Table S2). The detailed mutation information of the 196 patients is provided in Table S3.

**FIGURE 1:**
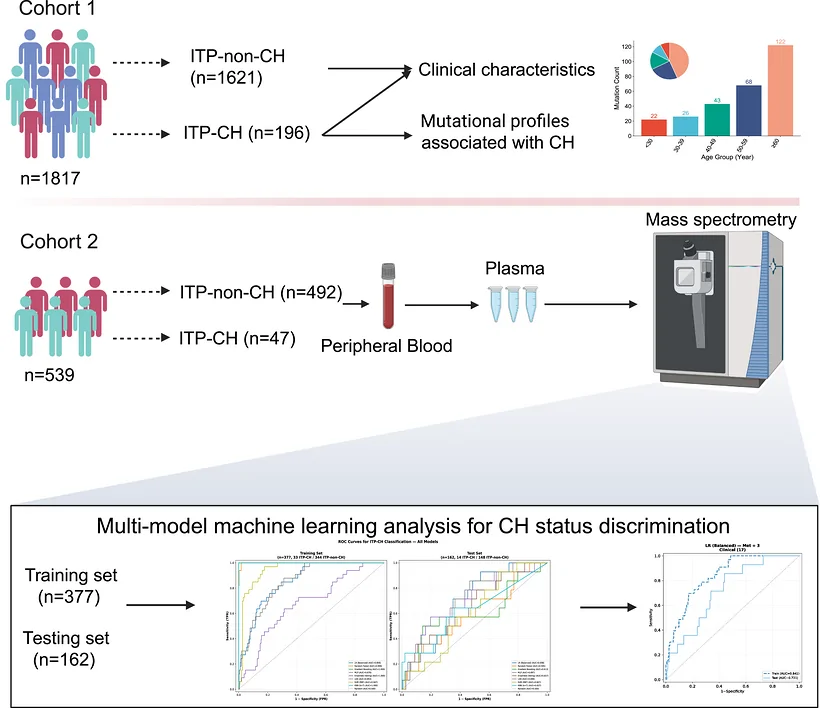
Overall design of the study. Cohort 1 included 1817 patients with chronic ITP; Cohort 2 included 539 patients with chronic ITP. ITP: immune thrombocytopenia; CH: clonal hematopoiesis.

**FIGURE 2:**
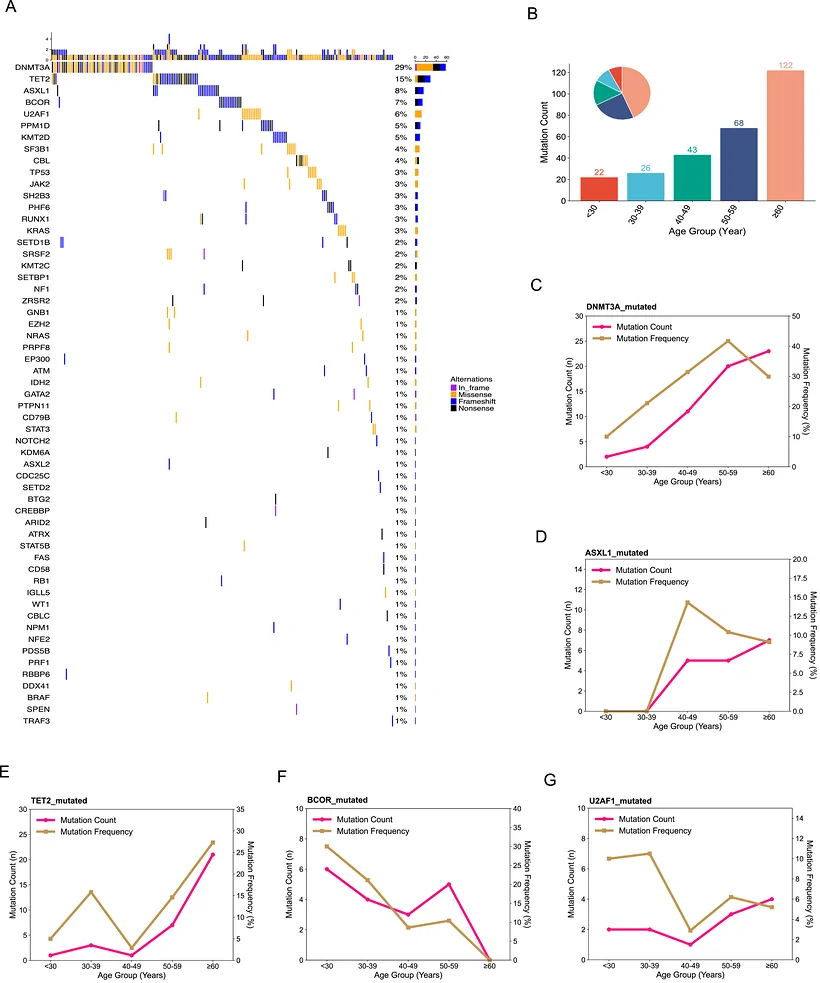
Landscape and age‐related distribution of clonal hematopoiesis (CH)‐associated mutations in ITP‐CH patients (cohort 1). (A) Oncoplot summarizing the mutational spectrum of 196 patients with immune thrombocytopenia (ITP). Each column represents an individual patient, and each row denotes a recurrently mutated gene. Mutations are color‐coded based on their type: in‐frame (dark blue), missense (orange), frameshift (yellow), and nonsense (light blue). The top bar indicates the number of mutations per patient, and the right bar displays the mutation frequency of each gene. *DNMT3A* was the most frequently mutated gene, followed by *TET2* and *ASXL1*. (B) Bar plot showing the total number of mutations stratified by age group (<30, 30–39, 40–49, 50–59, ≥60 years). A steep increase in mutation count is observed with advancing age, particularly in patients aged ≥60. The inset pie chart depicts the proportion of total mutations contributed by each age group. (C–G) Line plots illustrating the mutation count (pink line, left *y*‐axis) and mutation frequency (brown line, right *y*‐axis) of the five most frequently mutated genes across age groups: (C) *DNMT3A*, (D) *ASXL1*, (E) *TET2*, (F) *BCOR*, and (G) *U2AF1*.

### 2.2 Patient Characteristics and Mutational Profiles in Cohort 2

The cohort 2 included two subgroups: ITP‐non‐CH (*n* = 492), and ITP‐CH (*n* = 47), as detailed in Table S4. ITP‐CH patients were older than ITP‐non‐CH patients (median age: 59 years, IQR: 46–68 years vs. 45 years, IQR: 28.3‐57 years; *p* < 0.001). The mutation profile of ITP‐CH was shown in Figure 3 and Table S5. *DNMT3A* mutations were the most frequent (47%), followed by *TET2* (19%), *BCOR* (11%), *ASXL1* (6%), and *ZRSR2* (4%).

**FIGURE 3:**
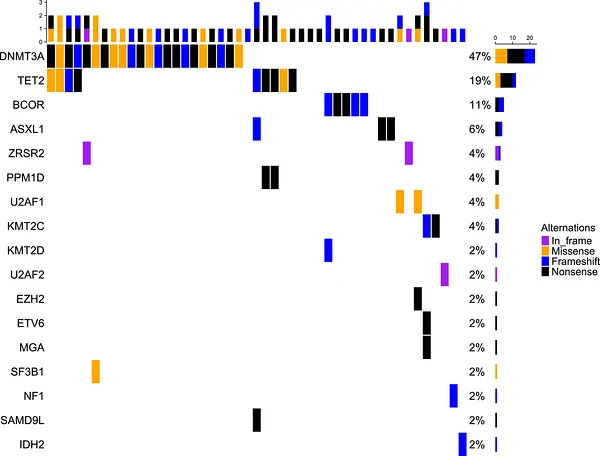
Mutational landscape of clonal hematopoiesis in patients with immune thrombocytopenia (ITP) from Cohort 2. This oncoplot summarizes the spectrum and frequency of recurrent gene mutations identified in ITP patients with clonal hematopoiesis (*n* = 47). Each column represents an individual patient, and each row corresponds to a gene harboring a mutation in at least 2% of the cohort. The color of each cell denotes the type of mutation observed, with in‐frame mutations shown in purple, missense in orange, frameshift in blue, and nonsense in black, as indicated in the legend. Mutation frequency (%) for each gene is listed to the right of the heatmap. The horizontal bar plot at the top displays the number of mutations per patient, indicating the mutational burden across individuals.

### 2.3 Metabolic Signatures Varied Significantly According to Genetic Mutation Classification

To further investigate the impact of genetic mutation types on metabolic profiles, we categorized gene functions into three major classes: epigenetic regulation, transcription/chromatin remodeling, and RNA splicing (each group containing greater than or equal to three patients). We utilized orthogonal partial least squares discriminant analysis (OPLS‐DA) to differentiate groups according to their metabolic profiles. After normalizing the data, we established an OPLS‐DA model that demonstrated clear and robust separation among these three groups (Figure S2A,7 Table S6), demonstrating significant inter‐group metabolic heterogeneity. Differential analysis identified that the majority of the top 20 differentially expressed metabolites were upregulated in patients with epigenetic regulation gene mutations (Figure S2B,D,F). Functional enrichment analysis highlighted significant involvement of gluconeogenesis, Warburg effect, and ketone body metabolism pathways (Figure S2C,E,G).

### 2.4 Metabolic Characteristics in *DNMT3A*‐R882‐Mutant CH

As somatic mutation at the *DNMT3A* R882 hotspot represents the most frequent molecular etiology of CH and is related to increased myeloid neoplasm risk [10, 13, 14], we conducted a comparative metabolomic analysis between R882 versus non‐R882 mutations in patients with *DNMT3A* mutations. OPLS‐DA demonstrated clear metabolic segregation between the two groups (Figure 4A). Detailed differential metabolites are shown in Table S7. Among the top 20 differentially abundant metabolites, Val‐Gln, PE (20:2(11Z,14Z)/18:2(9Z,12Z)), and diethyl glutarate were significantly elevated in the R882 cohort, while other metabolites showed higher abundance in non‐R882 carriers (Figure 4B). Functional enrichment analysis of differential metabolites identified significant pathway perturbations, including α‐linolenic and linoleic acid metabolism, Betaine metabolism, Methionine metabolism, and Ketone body metabolism (Figure 4C).

**FIGURE 4:**
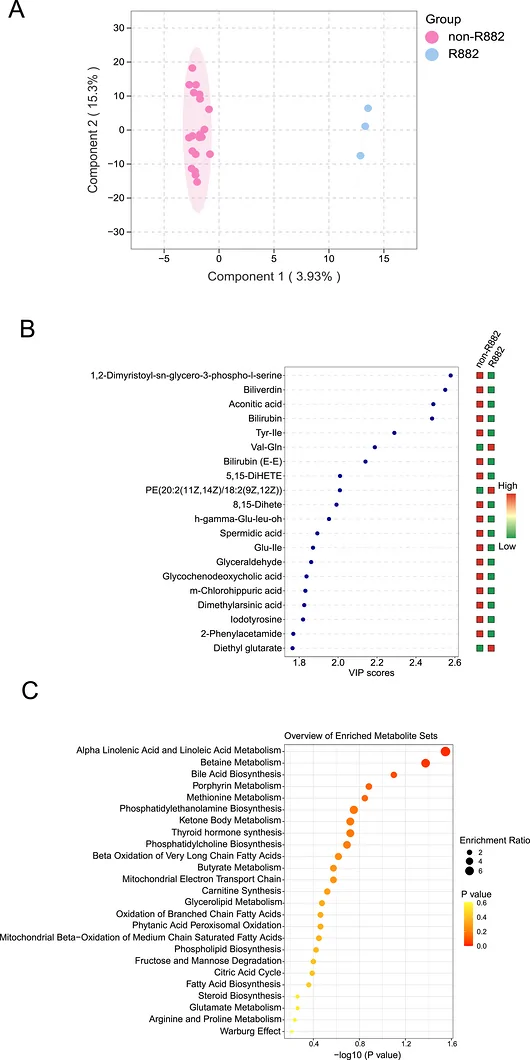
Metabolic clustering analysis and significance of differential metabolites between patients with *DNMT3A*‐R882 and *DNMT3A*‐non‐R882 mutated clonal hematopoiesis. (A) This heatmap illustrates the various metabolic express patterns between *DNMT3A*‐R882 and *DNMT3A*‐non‐R882 mutated patients. (B) The VIP scores range from 1.8 to 2.6, with higher scores indicating greater importance in the model. A color gradient from blue to red represents the mean expression levels, with blue indicating low expression and red indicating high expression. Metabolites such as “1,2‐dimyristoyl‐sn‐glycero‐3‐phospho‐l‐serine” and “bilirubin” are marked with red squares, indicating both high VIP scores and high expression levels. (C) This bubble plot provides an overview of enriched metabolic sets. The *x*‐axis represents the −log10(*p*‐value), indicating the statistical significance of enrichment, with higher values indicating greater significance. The *y*‐axis lists various metabolic pathways, with the size of the bubbles representing the enrichment ratio (ratio of observed to expected metabolites in the pathway). The color gradient from yellow to red represents the *p*‐value, with red indicating lower *p*‐values and thus higher significance.

### 2.5 Multi‐Model Machine Learning Analysis for CH Status Discrimination

To evaluate the discriminative capacity of integrated metabolic‐clinical features for CH status, we identified 14 differentially abundant plasma metabolites between ITP‐non‐CH and ITP‐CH patients (Figure S3, Table S8). These metabolites, combined with five clinical features (age, sex, PLT, ANC, Hb), constituted a 19‐feature input vector. The cohort was randomly partitioned into a training set (*n* = 377; 33 ITP‐CH, 344 ITP‐non‐CH) and an independent test set (*n* = 162; 14 ITP‐CH, 148 ITP‐non‐CH) using stratified sampling (7:3 ratio). Eight machine learning algorithms were systematically evaluated (Methods, Table S9). Three feature configurations were compared: (i) 14 metabolites only, (ii) 14 metabolites + 3 clinical features (sex, age, PLT), and (iii) 14 metabolites + 5 clinical features (sex, age, PLT, ANC, Hb). All features were standardized to zero mean and unit variance using training set parameters.

Among these models, logistic regression with balanced class weighting demonstrated consistent CH detection capability across all configurations. The optimal configuration, 14 metabolites combined with three clinical features (sex, age, PLT), achieved the best test performance (AUC = 0.721, recall = 0.429, specificity = 0.764, *F*1 = 0.218; Table S9), correctly identifying 6 of 14 ITP‐CH cases in the test set. The training set confusion matrix showed TP = 26, TN = 251, FP = 93, FN = 7 (accuracy: 0.735, AUC: 0.841). The train‐test AUC gap of 0.120 indicates modest overfitting, substantially less than that observed for tree‐based models (RF, GBM, KNN; gap > 0.35).

We found that adding ANC and Hb to the model (14 Met + 5 clinical, 19 features) did not improve performance (test AUC: 0.698 vs 0.721 for 14 Met + 3 clinical), indicating that these two hematologic parameters do not contribute incremental predictive information for CH discrimination beyond that already captured by sex, age, and PLT. The metabolites‐only model (AUC = 0.599) underperformed all configurations containing clinical features, confirming that clinical variables carry substantial CH‐related signal that cannot be captured by metabolomic data alone.

Aggregate feature importance analysis across tree‐based models and logistic regression coefficients identified age, undecanedioic acid, LPE (20:3/0:0), *N*‐acetyl‐D‐glucosamine, glycocholic acid, and carnitine C14:2 as the most discriminative features. Age was the dominant predictor (|*β*| = 0.351 in the LR model), consistent with the well‐established age‐dependence of clonal hematopoiesis. ROC curves for all model configurations on both training and test sets are presented in Figure 5. Complete model comparison data for all eight algorithms are provided in Table S9.

**FIGURE 5:**
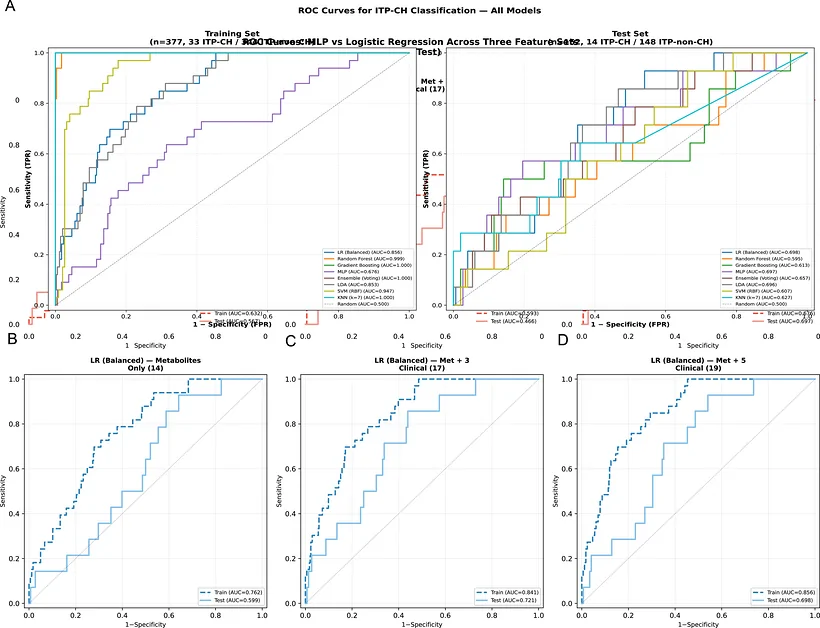
Multi‐model comparison and logistic regression (LR) model performance. (A) ROC curves of all 8 machine learning models on the train and test sets. (B) ROC curves of LR across three feature configurations: 14 metabolites only, 14 Met + 3 Clin, and 14 Met + 5 Clin.

To facilitate potential future research applications, we provide the complete logistic regression probability formula based on the optimal 17‐feature configuration (14 metabolites + sex, age, PLT). The estimated probability of ITP‐CH is given by:

$$P\left(\text{ITP}-\text{CH}\right)=1/\left(1+e^{-z}\right)$$

$$z=-0.386434+\Sigma_{i=1}17\;\beta_{i}\times \left(X_{i}-\mu_{i}\right)/\sigma_{i}$$

where *β_i_*, *µ_i_*, and *σ_i_* for all 17 features are provided in Table S10. The complete formula enables independent researchers to compute CH probability estimates for external validation cohorts. This formula is provided solely as an exploratory hypothesis‐generating tool.

## 3 Discussion

To our knowledge, this study represents the first investigation to characterize the metabolic profiles of ITP patients with or without gene mutations related to CH, leveraging a large clinical cohort and plasma metabolomics. We further systematically evaluated the discriminative capacity of integrated clinical‐metabolic features through eight machine learning algorithms to assess their potential to differentiate between ITP patients with and without CH‐related mutations. Our findings suggest distinct patterns of hematologic‐metabolic regulation between these groups, and logistic regression with 14 metabolites and three clinical features achieved the best discriminative balance, though with limited sensitivity for CH detection. This work provides a preliminary foundation and generates hypotheses for future mechanistic and clinical studies exploring the role of clonal hematopoiesis in ITP and related benign hematological conditions.

Clonal hematopoiesis of indeterminate potential (CHIP) is well‐established as a precursor to hematologic malignancies, especially myeloid neoplasms [15, 16]. In contrast, its role in benign hematologic disorders remains poorly characterized. A recent study reported that CHIP was related with an increased risk of ITP in a large cohort [17]. Current evidence suggests that ITP‐CH experience poorer treatment responses and higher rates of adverse events, including bleeding complications, indicating a less favorable prognosis compared to CH‐negative ITP cases [8]. Notably, differentiating between ITP‐non‐CH and ITP‐CH remains clinically challenging, especially in cytopenic patients and in settings where NGS is not readily available.

Mutations in DNMT3A and TET2, which encode epigenetic regulators, are the most prevalent in CHIP. DNMT3A loss‐of‐function mutations confer a competitive advantage to HSPCs by promoting self‐renewal bias, particularly under inflammatory stress [18, 19]. DNMT3A‐mutant HSPCs exhibit impaired lymphoid differentiation and preferential myeloid skewing, thereby reducing erythroid and megakaryocytic output [18, 20]. TET2‐deficient HSPCs not only expand clonally but also generate progeny macrophages that secrete excess interleukin‐1β (IL‐1β) and IL‐6, creating a feedback loop that further destabilizes hematopoiesis [18, 20]. Mouse models demonstrate that TET2 loss in myeloid progenitors directly contributes to thrombocytopenia [18]. Mutations in spliceosome genes (SF3B1, SRSF2, U2AF1, ZRSR2) disrupt pre‐mRNA splicing, leading to the accumulation of mis‐spliced transcripts and proteotoxic stress in hematopoietic progenitors [20, 21]. These defects trigger apoptosis in megakaryocytic lineages, manifesting as thrombocytopenia.

Our previous study revealed that metabolomics could serve as a valuable tool in understanding the complex pathophysiology of ITP and holds promise for predicting response to therapy [12]. In this study, we applied untargeted metabolomic profiling to characterize the plasma metabolic landscape of chronic ITP patients with and without CH. Our data reveal distinct metabolic signatures associated with CH status. These signatures align with emerging concepts of metabolic reprogramming in clonal hematopoiesis, as reported in other biological contexts. Prior studies [22, 23, 24, 25, 26], primarily in model systems, indicate that deficiencies in epigenetic regulators such as TET2 are associated with a shift in hematopoietic stem cell metabolism toward oxidative phosphorylation, which may alter redox homeostasis and cell fate. Similarly, mutations in DNMT3A, particularly the R882 variant, have been linked to adaptations in mitochondrial metabolism. Our observations are consistent with this broader framework. We found that ITP patients carrying CH‐related mutations, especially in epigenetic modifier genes, exhibited metabolic profiles enriched for pathways involved in mitochondrial energy metabolism, gluconeogenesis, and ketone body metabolism. This suggests a potential convergence on a metabolic state that could support clonal fitness by meeting anabolic demands and mitigating redox stress [27, 28, 29]. Notably, several metabolites that distinguished DNMT3A‐R882 from non‐R882 ITP‐CH cases are implicated in mitochondrial energy homeostasis and redox regulation [29, 30, 31, 32, 33], including those within α‐linolenic acid metabolism and betaine/methionine cycles. These findings are congruent with recent evidence suggesting that DNMT3A‐R882 mutations may enhance oxidative phosphorylation in hematopoietic progenitors, promoting clonal expansion through mitochondrial dependence [10]. Intriguingly, preclinical studies propose that such mitochondrial dependence might represent a therapeutic vulnerability; for instance, metformin has been shown to suppress DNMT3A‐R882‐mutant clones in experimental models [10]. Collectively, these observations demonstrate that the presence of CH in ITP may be associated with a detectable and specific remodeling of the plasma metabolome. This provided the rationale for developing a predictive model to distinguish ITP patients based on CH status.

Building on the clinical, hematologic, and metabolic alterations associated with CH, we integrated these multi‐modal features and systematically compared eight machine learning algorithms. Logistic regression with 14 metabolites combined with sex, age, and platelet count achieved the best discriminative balance. Because age, sex, and platelet count were included as covariates, the metabolic associations captured by the model are less likely to reflect confounding by these demographic and disease‐activity factors and more likely to reflect CH‐specific biology. The most discriminative metabolites are also biologically plausible: undecanedioic acid and carnitine C14:2 are intermediates of fatty acid oxidation, glycocholic acid is a bile acid, and *N*‐acetyl‐D‐glucosamine is a product of hexosamine metabolism. These features converge on mitochondrial energy metabolism and nutrient sensing, consistent with the metabolic reprogramming toward oxidative phosphorylation reported in clonal hematopoiesis [10, 22, 23, 24, 25, 26]. The model showed high specificity for excluding CH (76.4%) but limited sensitivity for CH detection (42.9%), a profile that favors ruling out CH over case finding. This signature should therefore be regarded as a preliminary, exploratory panel that requires external validation before any clinical application; if validated, it could serve as a risk‐stratification aid to prioritize confirmatory genomic testing.

Our systematic comparison across eight algorithms and three feature configurations offers additional insights. The multilayer perceptron collapsed to a trivial predict‐all‐negative strategy under extreme class imbalance (10.5:1), illustrating that deep learning does not by itself overcome the statistical challenge of rare‐event prediction when the minority class contains only 33 training examples. Logistic regression with balanced class weighting consistently generalized better than more complex models (random forest, gradient boosting, and support vector machine). This pattern is consistent with simulation evidence showing that flexible machine‐learning models are data‐hungry and prone to overfitting when the effective sample size is small [34]. The optimal 17‐feature configuration (14 metabolites + sex, age, PLT) achieved a test AUC of 0.721, correctly identifying 6 of 14 CH cases.

The marked inter‐individual metabolic heterogeneity within the ITP‐non‐CH group offers a biological explanation for the limited sensitivity. Human plasma metabolomes vary substantially between individuals as a result of genetic, dietary, and environmental factors [35]; this variation produces a broad metabolic baseline against which the relatively subtle metabolic shifts associated with CH are difficult to resolve. From a clinical standpoint, this finding tempers expectations for metabolomics‐based CH screening: the intrinsic variability of the human metabolome may impose a ceiling on achievable classification accuracy for this task, regardless of algorithmic sophistication.

This study has several limitations. First, we could not determine whether the association between ITP‐CH and CCUS reflects true coexistence or sequential disease progression; although bone marrow pathology confirmed ITP in our cohort (excluding bone marrow failure, MDS, or tumor infiltration), the overlapping clinical features of these entities highlight the need for multi‐omics approaches to distinguish ITP with versus without CH more precisely. Second, we excluded CCUS cases presenting with only non‐thrombocytopenic cytopenia, which limited our ability to compare their clinical and biological characteristics with those of ITP‐CH. Third, the absence of long‐term follow‐up and detailed documentation of adverse events or clonal evolution in ITP‐CH patients underscores the need for prospective studies to assess the risk of disease progression. Fourth, we lacked metabolomic data from patients on active therapy, precluding assessment of how treatment modifies CH‐associated metabolic signatures and influences biomarker development. Fifth, the logistic regression model and its probability formula require external validation in multicenter, prospective cohorts to establish generalizability and clinical utility. Sixth, all models showed limited sensitivity for CH detection across all feature configurations, suggesting that plasma metabolomics alone may have inherent ceiling effects for this classification task. Seventh, the logistic regression probability formula has not undergone external validation and must not be used for clinical decision‐making; NGS remains the gold standard for CH confirmation. Eighth, the MLP model was non‐reproducible across different random initializations, which further supports the choice of logistic regression because of its deterministic and reproducible nature.

In summary, we characterized the mutational landscape of ITP patients with and without CH and delineated the plasma metabolic profiles associated with CH status. Through systematic multi‐model machine learning, we provide a transparent logistic regression probability formula as an exploratory, hypothesis‐generating tool, while explicitly acknowledging that external validation is required and that NGS remains the gold standard for CH detection.

## 4 Materials and Methods

### 4.1 Patients

This investigation employed a retrospective study conducted at the Institute of Hematology and Blood Diseases Hospital (CAMS & PUMC, Tianjin, China). Figure 1 presents the overall design and methodological framework. The study comprised two independent cohorts. For both cohorts, the diagnosis of chronic ITP was defined as sustained thrombocytopenia persisting for more than 12 months [36] and was applied retrospectively after confirming the disease course through follow‐up. Patients were excluded if their thrombocytopenia was determined to be secondary to other conditions, including but not limited to: autoimmune diseases (e.g., systemic lupus erythematosus), chronic viral infections (hepatitis B, hepatitis C, HIV), lymphoproliferative disorders, confirmed *Helicobacter pylori* infection, or use of known thrombocytopenic medications. (1) Cohort 1 (Genomic Discovery Cohort): Included 1817 patients meeting the above diagnostic criteria, who were enrolled before December 1, 2021; (2) Cohort 2 (Metabolomic Profiling Cohort): Included 539 patients meeting the same diagnostic criteria, enrolled between December 1, 2021 and November 30, 2024. A defining feature of this cohort is that all plasma samples used for metabolomic analysis were required to have been collected during a verified treatment‐free period: specifically, when the patient was either treatment‐naïve or had been off all ITP‐specific therapies for at least 3 months.

Diagnostic criteria for ITP with clonal hematopoiesis (ITP‐CH): (1) Patients with a diagnosis of ITP according to the internationally diagnostic criteria [36]; (2) presence of clonal hematopoiesis‐related gene mutations with a variant allele frequency (VAF) between 2% and 40%; (3) the study population comprised patients with documented treatment responsiveness to corticosteroids and/or intravenous immunoglobulin (IVIG) therapy. (4) None of the patients exhibited sustained erythrocytosis, leukocytosis, thrombocytosis, splenomegaly, or constitutional symptoms suggestive of MPN during baseline evaluation. Bone marrow examinations showed no evidence of morphologic dysplasia, blast, or fibrosis compatible with WHO/ICC‐defined MPN or MDS.

The study was carried out in accordance with the ethical standards of the Declaration of Helsinki. The study was approved by the Ethics Committee of the Institute of Hematology & Blood Diseases Hospital, Chinese Academy of Medical Sciences & Peking Union Medical College (Approval Number: IIT2021031‐EC‐1), and written informed consent was obtained from all participants.

### 4.2 Next‐Generation Sequencing (NGS)

Bone marrow mononuclear cell DNA from two cohorts at diagnosis underwent NGS using the NovaSeq 6000 platform (Illumina), achieving a minimum mean sequencing depth of 800× as previously described. Two sequential gene panel configurations were implemented: a 141‐gene hematologic malignancy‐focused panel (Table S11) from August 2016 to March 2020, subsequently upgraded to a 267‐gene panel (Table S12) spanning April 2020 to July 2022. A multi‐tiered bioinformatics framework was deployed for systematic mutation profiling: Population frequency filtering against dbSNP [37], 1000 Genomes Project [38], and ESP6500 databases (http://evs.gs.washington.edu) to exclude polymorphisms (minor allele frequency >0.1%); Functional annotation of coding variants using computational predictors including SIFT (http://sift‐dna.org) and PolyPhen‐2 (http://genetics.bwh.harvard.edu/pph2) to assess protein‐level consequences; Pathogenicity prioritization through integrative analysis of COSMIC cancer mutation signatures [39], ACMG/AMP classification criteria, literature evidence, and an institutional clinical genomics repository, establishing a multidimensional classification system for identifying pathogenic or likely pathogenic variants.

### 4.3 Definition of CH

For variant calling, we established a minimum VAF threshold of 1%. CH was specifically defined by variants meeting or exceeding 2% VAF. Additionally, to minimize germline contamination, variants with VAF values surpassing 40% were systematically filtered out [8]. Somatic mutations identified from targeted sequencing were annotated using standard variant calling pipelines. To facilitate downstream analysis, mutated genes were functionally categorized into three groups based on previously published definitions: [40, 41] (1) Epigenetic regulators (e.g., *DNMT3A, TET2, ASXL1*); (2) Transcription/chromatin remodeling factors (e.g., *RUNX1, TP53, CUX1*); (3) RNA splicing factors (e.g., *SF3B1, SRSF2, U2AF1*).

### 4.4 Plasma Isolation and Metabolomics Analysis

Plasma samples from all patients in cohort 2 were collected for metabolomic analysis. Metabolomic profiling of plasma samples was conducted for treatment‐naïve patients or those with a treatment withdrawal period of at least 3 months. Details of sample preparation, metabolite extraction, mass spectrometry, and LC‐MS analysis were reported in our previous study [12]. Details of bioinformatic analysis are provided in the Supplementary Methods.

### 4.5 Multi‐Model Machine Learning Analysis

The 14 differentially expressed metabolites (VIP > 1, fold change > 1.2 or < 0.8, *p* < 0.05) and clinical features constituted the input vectors. Three feature configurations were evaluated: (i) 14 metabolites only; (ii) 14 metabolites + sex, age, PLT (17 features); and (iii) 14 metabolites + sex, age, PLT, ANC, Hb (19 features). All features were standardized using *Z*‐score normalization based on the training set parameters. The cohort was randomly partitioned into training (*n* = 377, 70%) and test (*n* = 162, 30%) sets using stratified sampling. Eight algorithms were evaluated: (1) logistic regression (L2 regularization, *C* = 0.01, class_weight = “balanced”, solver = “saga”, max_iter = 5000); (2) random forest (500 trees, max_depth = 5, min_samples_split = 10, class_weight = “balanced”); (3) gradient boosting (200 trees, max_depth = 3, learning_rate = 0.05); (4) multilayer perceptron (two hidden layers: 128 and 64 neurons, ReLU, batch normalization, dropout = 0.5, early stopping, batch_size = 32); (5) support vector machine (RBF kernel, *C* = 0.5, class_weight = “balanced”); (6) linear discriminant analysis (eigenvalue shrinkage); (7) K‐nearest neighbors (*k* = 7, distance‐weighted); and (8) soft‐voting ensemble (LR + RF + GBM + LDA, weights = 1:2:2:1). Model performance was assessed using accuracy, *F*1‐score, precision, recall, and AUC. Feature importance was aggregated across tree‐based models (Gini importance) and linear models (absolute standardized coefficients). The logistic regression probability formula was derived from the optimal 17‐feature model (14 metabolites + sex, age, PLT). All analyses used scikit‐learn 1.3 in Python 3.7.

### 4.6 Statistical Analysis

All statistical analyses were performed using SPSS version 26.0 (IBM Corp.), R version 4.2.1, and Python version 3.7. Continuous data are summarized as medians with interquartile ranges (IQR), whereas categorical data are reported as counts and percentages. Group comparisons were assessed using the chi‐square test or Fisher's exact test, as appropriate. A two‐sided *p*‐value of less than 0.05 was considered statistically significant.

## Author Contributions

J.C., Q.W., and T.S. contributed equally to this study as co‐first authors. L.Z., Y.F.C, and J.C. contributed to the design of the study. J.C. contributed to the analysis and interpretation of the data and the preparation of figures for the manuscript. J.C., Q.W., and T.S. contributed to the literature search of the study and wrote the manuscript. X.F.L., F.X., W.L., R.F.F., M.K.J., Y.C., W.T.W., and R.C.Y. contributed to the collection of the study. All authors had full access to all the data in the study and approved the final version of the manuscript.

## Funding

This work was supported by grants from the Noncommunicable Chronic Diseases‐National Science and Technology Major Project (2023ZD0500803), CAMS Innovation Fund for Medical Sciences (CIFMS) (2025‐I2M‐C&T‐B‐079; 2024‐I2M‐C&T‐B‐084), National Key Research and Development Program of China (2023YFC2507803), National Natural Science Foundation of China (82470136), and Tianjin Natural Science Foundation Project (25JCYBJC00330).

## Conflicts of Interest

The authors declare no conflicts of interest.

## Ethics Statement

The study was approved by the Ethics Committee of the Institute of Hematology & Blood Diseases Hospital, Chinese Academy of Medical Sciences & Peking Union Medical College (Approval Number: IIT2021031‐EC‐1). Written informed consent was obtained from all participants.

## Supporting information




**Supporting Information**: mco271129‐sup‐0001‐SuppMat.docx

## Data Availability

The data that support the findings of this study are available from the corresponding author (Lei Zhang, zhanglei1@ihcams.ac.cn) upon reasonable request.
