# Supplementary material for: Metabolic Profiling Combined With Machine Learning for Assessing Clonal Hematopoiesis Status in Chronic Immune Thrombocytopenia: An Exploratory Analysis
Source: MedComm (2020). 2026 Nov 8;7(11):e71129. doi: 10.1002/mco2.71129 (PMC13744647; doi:10.1002/mco2.71129)
Supplement: Supplementary file 1 [file MCO2-7-e71129-s001.docx]

Supplementary Materials for

**Metabolic Profiling Combined with Machine Learning for Assessing**

**Clonal Hematopoiesis Status in Chronic Immune Thrombocytopenia:**

**An Exploratory Analysis**

Jia Chen^1,2^#, Qing Wen^1,2^#, Ting Sun^1,2^#, Xiaofan Liu^1,2^, Feng Xue^1,2^, Wei Liu^1,2^, Rongfeng Fu^1,2^, Mankai Ju^1,2^, Xinyue Dai^1,2^, Ying Chi^1,2^, Wentian Wang^1,2^, Renchi Yang^1,2^, Yunfei Chen^1,2^*, Lei Zhang^1,2,3^*

Correspondence to: Lei Zhang, M.D., [zhanglei1@ihcams.ac.cn](mailto:zhanglei1@ihcams.ac.cn)

Yunfei Chen, M.D., chenyunfei@ihcams.ac.cn

**Materials and Methods**

**1. Sample preparation and extraction 6.1.1.1 Liquid samples class I**

The sample stored at -80 °C refrigerator was thawed on ice and vortexed for 10 s. 50 μL of sample and 300 μL of extraction solution (ACN: Methanol = 1:4, V/V) containing internal standards were added into a 2 mL microcentrifugetube. The sample was vortexed for 3 min and then centrifuged at 12000 rpm for 10 min (4 °C). 200 μL of the supernatant was collected and placed in -20 °C for 30 min, and then centrifuged at 12000 rpm for 3 min (4 °C). A 180 μL aliquots of supernatant were transferred for LC-MS analysis.

**2. HPLC Conditions**

All samples were for two LC/MS methods. One aliquot was analyzed using positive ion conditions and was eluted from T3 column (Waters ACQUITY Premier HSS T3 Column 1.8 μm, 2.1 mm * 100 mm) using 0.1 % formic acid in water as solvent A and 0.1 % formic acid in acetonitrile as solvent B in the following gradient: 5 to 20 % in 2 min, increased to 60 % in the following 3 mins, increased to 99 % in 1 min and held for 1.5 min, then come back to 5 % mobile phase B witnin 0.1 min, held for 2.4 min. The analytical conditions were as follows, column temperature, 40 °C; flow rate, 0.4 mL/min; injection volume, 4 μL; Another aliquot was using negative ion conditions and was the same as the elution gradient of positive mode.

**3. MS Conditions (QE)**

All the methods alternated between full scan MS and data dependent MSn scans using dynamic exclusion. MS analyses were carried out using electrospray ionization in the positive ion mode and negative ion mode using full scan analysis over m/z 75-1000 at 35000 resolutions. Additional MS settings are: ion spray voltage, 3.5 KV or 3.2 KV in positive or negative modes, respectively; Sheath gas (Arb), 30; Aux gas, 5; Ion transfer tube temperature, 320 °C; Vaporizer temperature, 300 °C; Collision energy, 30,40,50 V; Signal Intensity Threshold, 1*e6 cps; Top N vs Top speed, 10; Exclusion duration, 3s.

**4. Differential metabolites selected**

For two-group analysis, differential metabolites were determined by VIP >1, fold change >1.2 or <0.8, and *P* < 0.05 (Student’s t test). VIP values were extracted from OPLS-DA result, which also contain score plots and permutation plots, was generated using R package MetaboAnalystR. The data was log transform (log_2_) and mean centering before OPLS-DA. In order to avoid overfitting, a permutation test (200 permutations) was performed.

**5. KEGG annotation and enrichment analysis**

Identified metabolites were annotated using KEGG Compound database (http://www.kegg.jp/kegg/ compound/), annotated metabolites were then mapped to KEGG Pathway database (http://www.kegg.jp/ kegg/pathway.html).

**6. Multi-model machine learning analysis**

To systematically evaluate the discriminative capacity of integrated metabolic-clinical features, eight machine learning algorithms were trained and evaluated on the same training (n = 377) and test (n = 162) sets. Three feature configurations were evaluated: (i) 14 metabolites only (14 features), (ii) 14 metabolites + 3 clinical features (sex, age, PLT; 17 features), and (iii) 14 metabolites + 5 clinical features (sex, age, PLT, ANC, Hb; 19 features). The optimal configuration was 14 metabolites + 3 clinical features (17 features). All features were standardized: X_std = (X_raw − μ) / σ, where μ and σ were computed from the training set (n = 377). The same μ and σ were applied to the test set to prevent data leakage.

Algorithms Evaluated. (1) Logistic Regression: L2 regularization, C = 0.01, class_weight = "balanced", solver = "saga", max_iter = 5,000. (2) Random Forest: 500 trees, max_depth = 5, min_samples_split = 10, min_samples_leaf = 5, class_weight = "balanced". (3) Gradient Boosting: 200 trees, max_depth = 3, learning_rate = 0.05. (4) Multilayer Perceptron: two hidden layers (128, 64), ReLU, batch normalization, dropout = 0.5, early stopping, batch_size = 32. (5) Support Vector Machine: RBF kernel, C = 0.5, class_weight = "balanced". (6) Linear Discriminant Analysis: solver = "eigen", shrinkage = "auto". (7) K-Nearest Neighbors: k = 7, weights = "distance". (8) Soft-Voting Ensemble: LR + RF + GBM + LDA, weights = [1, 2, 2, 1].

Performance Metrics. Accuracy, F1-score, Precision, Recall, and AUC were computed on both training and test sets. All confusion matrices were verified — row/column sums match the respective sample sizes exactly (Supplementary Table S7).

Logistic Regression Probability Formula. The final model uses 14 metabolites + 3 clinical features (sex, age, PLT; 17 features total). The probability formula is:

P(ITP-CH) = 1 / (1 + e⁻ᶻ), where z = -0.386434 + Σᵢ₌₁¹⁷ βᵢ × (Xᵢ − μᵢ)/σᵢ

All parameters (βᵢ, μᵢ, σᵢ for all 17 features) are provided in Supplementary Table S8.

**Figure S1. Frequency and type distribution of *DNMT3A* mutation sites in patients with ITP-CH in cohort 1.**

**Figure S2. (A)** Orthogonal partial least squares discriminant analysis (OPLS-DA) differentiates groups according to their metabolic profiles. **(B)** displays a dot plot and expression heatmap of the top 20 differential metabolites (ranked by VIP score) between the epigenetic regulation and RNA splicing groups. **(C)** presents the KEGG pathway enrichment analysis of the differentially abundant metabolites identified in these two groups. **(D)** displays a dot plot and expression heatmap of the top 20 differential metabolites (ranked by VIP score) between the epigenetic regulation and transcriptional and chromatin remodeling groups. **(E)** presents the KEGG pathway enrichment analysis of the differentially abundant metabolites identified in these two groups. **(F)** displays a dot plot and expression heatmap of the top 20 differential metabolites (ranked by VIP score) between the transcriptional and chromatin remodeling and RNA splicing groups. **(G)** presents the KEGG pathway enrichment analysis of the differentially abundant metabolites identified in these two groups.

**Figure S3. (A)** displays a dot plot and expression heatmap of the 14 differential metabolites (ranked by VIP score) between the ITP and ITP-CH groups. **(B)** illustrates the expression changes of various metabolites between two classes (Class I and Class II) by showing their Log_2_ fold change values. Each dot in the plot represents a metabolite, positioned according to its secondary classification, with color and size indicating its primary classification and the significance of the *P* value, respectively. X-axis (horizontal): Represents the secondary classification of metabolites, with each color series representing a category of primary classification. Y-axis (vertical): Represents the Log_2_ fold change values, indicating the expression changes of metabolites in Class II relative to Class I. Positive values suggest upregulation, while negative values indicate downregulation. Color: Represents the primary classification of metabolites, with different colors corresponding to different metabolite categories (listed in the legend as GP, GL, FA, and their subcategories). Size: Represents the magnitude of the *P* value, with larger bubbles indicating smaller *P* value, thus greater statistical significance.

**Table S1. Baseline characteristics of all patients in cohort 1.**

| **Characteristics** | **ITP-non-CH (n=1621)** | **ITP-CH (n=196)** | ***P* value** |
| --- | --- | --- | --- |
| Age, year (median, IQR) | 44 (28, 56) | 57 (45.3, 66.8) | <0.001 |
| Sex, n (%)  Female  Male | 956 (59)  665 (41) | 124 (63.3)  72 (36.7) | 0.248 |
| WBC, ×10^9^/L (median, IQR) | 6.9 (5.1, 9.6) | 5.9 (4.1, 8.5) | <0.001 |
| RBC, ×10^12^/L (median, IQR) | 4.5 (3.9, 4.9) | 4.1 (3.3, 4.6) | <0.001 |
| Hb, g/L (median, IQR) | 131 (116, 145) | 124 (106.3, 136.8) | <0.001 |
| PLT, ×10^9^/L (median, IQR) | 14 (5, 27) | 17 (6, 28.8) | 0.107 |
| ANC, ×10^9^/L (median, IQR) | 4.3 (2.9, 6.8) | 3.7 (2.1, 5.8) | <0.001 |
| Neutrophils percentage, % (median, IQR) | 64.6 (55.4, 73.1) | 61.6 (49.9, 69.8) | <0.001 |
| LDH, U/L (median, IQR) | 217.9 (182.8, 270.7) | 211.1 (169.9, 266.6) | 0.089 |
| Most common previous treatment, no.(%) | n=1360 | n=155 |  |
| Glucocorticoids | 883 (64.9) | 88 (56.8) | 0.045 |
| rhTPO | 476 (35) | 53 (34.2) | 0.842 |
| IVIg | 389 (28.6) | 44 (28.4) | 0.955 |
| TPO-RAs | 218 (16) | 25 (16.1) | 0.974 |
| Danazol | 197 (14.5) | 28 (18.1) | 0.235 |
| Cyclosporine | 130 (9.6) | 17 (11) | 0.574 |

IQR: interquartile range; WBC: white blood cell count; RBC: red blood cell count; ANC: absolute neutrophil count; Hb: hemoglobin; PLT: platelet count; ANC: absolute neutrophil count; LDH: lactate dehydrogenase; CH: clonal hematopoiesis. IVIg: intravenous immunoglobulin; rhTPO: recombinant human thrombopoietin (TPO); TPO-RAs: TPO receptor agonists.

**Table S2. The frequency and type distribution of *DNMT3A* mutation sites in cohort 1.**

| **AA_Change** | **Frameshift** | **In-frame Indel** | **Missense** | **Nonsense** | **Percentage (%)** |
| --- | --- | --- | --- | --- | --- |
| p.R882H | 0 | 0 | 7 | 0 | 11.67 |
| p.R882C | 0 | 0 | 5 | 0 | 8.33 |
| p.R736C | 0 | 0 | 4 | 0 | 6.67 |
| p.R736H | 0 | 0 | 4 | 0 | 6.67 |
| p.R882S | 0 | 0 | 2 | 0 | 3.33 |
| p.R771X | 0 | 0 | 0 | 2 | 3.33 |
| p.F732delF | 0 | 2 | 0 | 0 | 3.33 |
| p.R635W | 0 | 0 | 2 | 0 | 3.33 |
| p.A791Gfs*7 | 1 | 0 | 0 | 0 | 1.67 |
| p.R771* | 0 | 0 | 0 | 1 | 1.67 |
| p.R803K | 0 | 0 | 1 | 0 | 1.67 |
| p.R899C | 0 | 0 | 1 | 0 | 1.67 |
| p.S770* | 0 | 0 | 0 | 1 | 1.67 |
| p.S770Ffs*7 | 1 | 0 | 0 | 0 | 1.67 |
| p.V341fs | 1 | 0 | 0 | 0 | 1.67 |
| p.W313* | 0 | 0 | 0 | 1 | 1.67 |
| p.W314X | 0 | 0 | 0 | 1 | 1.67 |
| p.W581X | 0 | 0 | 0 | 1 | 1.67 |
| p.W698* | 0 | 0 | 0 | 1 | 1.67 |
| p.R688H | 0 | 0 | 1 | 0 | 1.67 |
| p.R458Gfs*193 | 1 | 0 | 0 | 0 | 1.67 |
| p.A910P | 0 | 0 | 1 | 0 | 1.67 |
| p.G543C | 0 | 0 | 1 | 0 | 1.67 |
| p.A910V | 0 | 0 | 1 | 0 | 1.67 |
| p.C494Lfs*52 | 1 | 0 | 0 | 0 | 1.67 |
| p.C559* | 0 | 0 | 0 | 1 | 1.67 |
| p.E371Gfs*36 | 1 | 0 | 0 | 0 | 1.67 |
| p.E561Afs*17 | 1 | 0 | 0 | 0 | 1.67 |
| p.E774X | 0 | 0 | 0 | 1 | 1.67 |
| p.F732del | 0 | 1 | 0 | 0 | 1.67 |
| p.H355Tfs*52 | 1 | 0 | 0 | 0 | 1.67 |
| p.R320X | 0 | 0 | 0 | 1 | 1.67 |
| p.I310T | 0 | 0 | 1 | 0 | 1.67 |
| p.L653F | 0 | 0 | 1 | 0 | 1.67 |
| p.L723Pfs*56 | 1 | 0 | 0 | 0 | 1.67 |
| p.M880fs*1 | 1 | 0 | 0 | 0 | 1.67 |
| p.Q249Lfs*65 | 1 | 0 | 0 | 0 | 1.67 |
| p.Q573X | 0 | 0 | 0 | 1 | 1.67 |
| p.Q692* | 0 | 0 | 0 | 1 | 1.67 |
| p.W893* | 0 | 0 | 0 | 1 | 1.67 |

**Table S3. Details of gene mutation profiles associated with clonal hematopoiesis (CH) in ITP patients in cohort 1.**

| **Pt** | **Gene** | **AA_change** | **VAF (%)** |
| --- | --- | --- | --- |
| P1 | TET2 | p.Y899X | 3.2 |
| P2 | TP53 | p.R335L | 5.1 |
| P3 | DNMT3A | p.R635W | 37.8 |
| P4 | TET2 | p.Y899X | 3.0 |
| P5 | ASXL1 | p.E635Rfs*14 | 2.5 |
| P6 | JAK2 | p.G127D | 5.0 |
| P7 | TET2 | p.E1151* | 4.4 |
| P8 | DNMT3A | p.R736C | 11.0 |
| P9 | U2AF1 | p.Q157P | 13.4 |
| P10 | TET2 | p.R814C | 10.6 |
| P11 | DNMT3A | p.R736H | 13.0 |
| P12 | BCOR | p.Q717* | 4.0 |
| P13 | DNMT3A | p.R771* | 2.1 |
| P14 | DNMT3A | p.R882H | 24.8 |
| P15 | DNMT3A | p.G543C | 5.6 |
| P16 | BCOR | p.A1267Efs*68 | 5.3 |
| P17 | CBL | p.Y371S | 2.1 |
| P18 | SH2B3 | p.D485_W492del | 16.1 |
| P18 | TET2 | p.T1985Nfs*30 | 5.0 |
| P19 | NOTCH2 | p.G2363Tfs*4 | 2.4 |
| P20 | DNMT3A | p.L653F | 2.1 |
| P20 | SETD1B | p.Q1589Pfs*19 | 2.4 |
| P21 | DNMT3A | p.Q692* | 3.4 |
| P22 | KMT2D | p.D3061Qfs*7 | 4.0 |
| P23 | DNMT3A | p.W698* | 7.5 |
| P23 | TET2 | p.L1212S | 3.1 |
| P24 | TET2 | p.Y899X | 4.6 |
| P25 | TET2 | p.Q790X | 8.3 |
| P26 | GNB1 | p.K57E | 11.2 |
| P26 | TET2 | p.Q635* | 25.6 |
| P26 | SRSF2 | p.P95H | 17.0 |
| P27 | KDM6A | p.R1415X | 30.5 |
| P27 | PHF6 | p.K21Mfs*2 | 26.3 |
| P28 | KMT2C | p.R1237* | 2.5 |
| P29 | EZH2 | p.R566H | 2.7 |
| P30 | ASXL1 | p.E635Rfs*15 | 36.3 |
| P30 | U2AF1 | p.S34Y | 33.0 |
| P31 | DNMT3A | p.A791Gfs*7 | 3.4 |
| P32 | DNMT3A | p.C559* | 10.7 |
| P33 | BCOR | p.I1645Sfs*39 | 4.8 |
| P34 | DNMT3A | p.R882S | 2.3 |
| P35 | ASXL1 | p.E635Rfs*14 | 20.4 |
| P36 | BCOR | p.A1503Cfs*2 | 6.9 |
| P37 | SETD1B | p.Y1701Sfs*42 | 21.1 |
| P37 | SH2B3 | p.L347Afs*38 | 13.9 |
| P38 | RUNX1 | p.S424Afs*129 | 22.0 |
| P39 | DNMT3A | p.R736H | 2.7 |
| P40 | U2AF1 | p.S34F | 31.5 |
| P40 | NRAS | p.G12D | 12.0 |
| P41 | ASXL2 | p.D1188Sfs*7 | 4.2 |
| P41 | EZH2 | p.R690H | 2.8 |
| P41 | PRPF8 | p.D1598Y | 5.4 |
| P41 | SRSF2 | p.P96L | 2.4 |
| P41 | TET2 | p.S462Vfs*24 | 26.2 |
| P42 | PPM1D | p.E475Kfs*8 | 11.8 |
| P43 | SRSF2 | p.P95H | 37.1 |
| P43 | TET2 | p.F776Wfs*4 | 40.0 |
| P44 | KMT2C | p.R907* | 2.4 |
| P45 | PPM1D | p.E540Ffs*2 | 15.8 |
| P46 | ASXL1 | p.G646Wfs*11 | 4.8 |
| P47 | PHF6 | p.Y301X | 10.7 |
| P48 | ASXL1 | p.G646Wfs*11 | 4.8 |
| P49 | JAK2 | p.V617F | 3.0 |
| P50 | DNMT3A | p.S770Ffs*7 | 5.3 |
| P51 | DNMT3A | p.R899C | 3.4 |
| P52 | TET2 | p.Q685X | 9.2 |
| P53 | CDC25C | p.R386Pfs*14 | 30.6 |
| P54 | TP53 | p.V272M | 2.6 |
| P55 | SF3B1 | p.K666N | 2.3 |
| P56 | BCOR | p.K1104Sfs*9 | 21.9 |
| P57 | U2AF1 | p.S34Y | 25.8 |
| P58 | EP300 | p.S2407*fs*1 | 10.7 |
| P58 | DNMT3A | p.W314X | 26.5 |
| P58 | DNMT3A | p.R882C | 8.2 |
| P59 | DNMT3A | p.R803K | 2.0 |
| P60 | PPM1D | p.T537Hfs*2 | 39.9 |
| P60 | KMT2C | p.R1092* | 2.5 |
| P60 | U2AF1 | p.Q157P | 22.6 |
| P61 | SETD2 | p.S458Ffs*2 | 3.4 |
| P62 | U2AF1 | p.S34F | 6.4 |
| P63 | BTG2 | p.Q33* | 14.4 |
| P63 | CREBBP | p.S1680del | 11.5 |
| P63 | KMT2D | p.K1472Sfs*34 | 5.5 |
| P64 | TET2 | p.C1273* | 18.8 |
| P64 | DNMT3A | p.R882H | 24.0 |
| P65 | ASXL1 | p.E635Rfs*15 | 8.1 |
| P65 | TET2 | p.I1873T | 39.1 |
| P65 | TET2 | p.L532X | 39.1 |
| P66 | DNMT3A | p.W893* | 6.7 |
| P66 | DNMT3A | p.R635W | 15.1 |
| P67 | ASXL1 | p.E635Rfs*15 | 34.7 |
| P67 | TET2 | p.R544X | 19.8 |
| P68 | TET2 | p.R1216X | 2.0 |
| P69 | CBL | p.R420* | 10.2 |
| P70 | TET2 | p.G1282Vfs*82 | 3.7 |
| P70 | GNB1 | p.K57N | 7.8 |
| P71 | DNMT3A | p.R882H | 2.7 |
| P72 | DNMT3A | p.L723Pfs*56 | 12.2 |
| P73 | DNMT3A | p.R736H | 3.6 |
| P74 | DNMT3A | p.W313* | 2.3 |
| P74 | ASXL1 | p.E477* | 9.5 |
| P75 | SETBP1 | p.G870S | 38.6 |
| P75 | RUNX1 | p.L98Sfs*24 | 39.0 |
| P76 | PPM1D | p.R572X | 4.3 |
| P77 | ASXL1 | p.G646Wfs*12 | 11.0 |
| P77 | ARID2 | p.Y133* | 13.4 |
| P78 | ATRX | p.G51* | 2.2 |
| P79 | ATM | p.E1832Gfs*4 | 2.4 |
| P79 | ATM | p.E2409Nfs*12 | 3.0 |
| P80 | BCOR | p.R1480X | 7.7 |
| P81 | IDH2 | p.R140Q | 20.0 |
| P82 | DNMT3A | p.R736C | 2.5 |
| P83 | EP300 | p.S507Afs*3 | 7.4 |
| P84 | DNMT3A | p.Q249Lfs*65 | 5.7 |
| P85 | TP53 | p.R248W | 7.9 |
| P85 | SF3B1 | p.K700E | 37.3 |
| P86 | PHF6 | p.R116X | 5.0 |
| P87 | DNMT3A | p.A910V | 9.6 |
| P88 | SF3B1 | p.K700E | 39.9 |
| P88 | JAK2 | p.V617F | 15.8 |
| P89 | KMT2D | p.L935Ffs*23 | 36.9 |
| P90 | DNMT3A | p.F732delF | 11.5 |
| P90 | BCOR | p.K212*fs*1 | 32.0 |
| P91 | DNMT3A | p.R882C | 2.0 |
| P92 | JAK2 | p.V617F | 2.0 |
| P92 | U2AF1 | p.S34F | 31.9 |
| P92 | STAT5B | p.N642H | 2.0 |
| P93 | SH2B3 | p.I446Sfs*18 | 2.9 |
| P94 | JAK2 | p.V617F | 37.3 |
| P95 | NF1 | p.I679Dfs*21 | 2.0 |
| P96 | DNMT3A | p.R882S | 2.1 |
| P97 | KMT2D | p.S654Pfs*13 | 13.3 |
| P98 | SF3B1 | p.K700E | 2.1 |
| P98 | TET2 | p.H1380Y | 2.7 |
| P98 | ASXL1 | p.L497* | 11.5 |
| P99 | DNMT3A | p.H355Tfs*52 | 10.0 |
| P100 | FAS | p.N223*fs*1 | 3.7 |
| P100 | CD58 | p.R152* | 3.8 |
| P101 | TET2 | p.N1266S | 1.6 |
| P101 | PPM1D | p.R552* | 15.4 |
| P102 | DNMT3A | p.R736C | 2.3 |
| P103 | RB1 | p.E465Vfs*10 | 10.4 |
| P103 | BCOR | p.Y657X | 13.6 |
| P104 | GATA2 | p.M388_K389delMK | 11.9 |
| P104 | SETBP1 | p.D868N | 34.5 |
| P105 | IGLL5 | p.M1L | 9.2 |
| P106 | WT1 | p.A382Gfs*69 | 7.2 |
| P106 | KRAS | p.G13D | 10.2 |
| P107 | PPM1D | p.N512Ifs*2 | 2.9 |
| P107 | SF3B1 | p.K700E | 8.0 |
| P108 | PHF6 | p.F263Lfs*16 | 38.2 |
| P109 | DNMT3A | p.A910P | 2.1 |
| P110 | DNMT3A | p.R771X | 4.1 |
| P111 | IDH2 | p.R172K | 0.8 |
| P111 | RUNX1 | p.R162S | 23.1 |
| P111 | ASXL1 | p.I814Ffs*4 | 22.3 |
| P111 | RUNX1 | p.R107C | 4.8 |
| P112 | CBLC | p.W12* | 2.3 |
| P113 | KRAS | p.A146T | 3.9 |
| P114 | KMT2D | p.K4556Rfs*19 | 26.4 |
| P114 | NPM1 | p.W288Cfs*12 | 30.0 |
| P114 | GATA2 | p.P274Tfs*8 | 10.4 |
| P115 | DNMT3A | p.W581X | 3.6 |
| P116 | BCOR | p.V1504Cfs*3 | 18.8 |
| P117 | SETD1B | p.Y32* | 4.4 |
| P117 | NFE2 | p.P79Lfs*32 | 29.8 |
| P118 | ASXL1 | p.L775* | 2.0 |
| P119 | PTPN11 | p.D61G | 6.7 |
| P120 | SETBP1 | p.D868N | 7.8 |
| P120 | PRPF8 | p.H1875R | 28.2 |
| P121 | TET2 | p.Y1598Ifs*12 | 37.1 |
| P122 | PPM1D | p.R552* | 5.5 |
| P122 | BCOR | p.M265*fs*1 | 2.6 |
| P123 | TET2 | p.H1732Tfs*13 | 38.0 |
| P124 | TP53 | p.Y220C | 2.2 |
| P125 | RUNX1 | p.S322Nfs*232 | 4.2 |
| P125 | ASXL1 | p.E727X | 7.1 |
| P126 | CD79B | p.Y196S | 3.0 |
| P126 | TET2 | p.D527Efs*6 | 3.9 |
| P127 | TET2 | p.Q1466X | 9.0 |
| P128 | DNMT3A | p.R882H | 3.8 |
| P129 | BCOR | p.I276Rfs*101 | 17.9 |
| P130 | NF1 | p.R1968X | 4.7 |
| P131 | DNMT3A | p.R736H | 3.3 |
| P132 | TET2 | p.L567Gfs*14 | 24.7 |
| P132 | SH2B3 | p.R202Qfs*64 | 2.4 |
| P133 | BCOR | p.W791* | 7.7 |
| P134 | U2AF1 | p.S34F | 36.5 |
| P134 | RUNX1 | p.P383Afs*173 | 39.1 |
| P134 | PHF6 | p.K72Rfs*9 | 13.4 |
| P135 | ATM | p.N1000Kfs*10 | 28.9 |
| P135 | SH2B3 | p.Q524X | 6.1 |
| P136 | BCOR | p.Y1464X | 12.7 |
| P137 | TP53 | p.R175H | 2.9 |
| P138 | DNMT3A | p.R882C | 2.3 |
| P139 | CBL | p.S767* | 2.2 |
| P140 | KMT2D | p.Q3826Cfs*3 | 24.2 |
| P140 | TET2 | p.R1465* | 2.9 |
| P141 | TP53 | p.E258K | 6.5 |
| P142 | DNMT3A | p.R320X | 3.6 |
| P143 | DNMT3A | p.R882H | 3.0 |
| P144 | SF3B1 | p.K700E | 9.8 |
| P145 | PDS5B | p.K1318Nfs*76 | 3.8 |
| P146 | ZRSR2 | p.Q56X | 5.4 |
| P146 | TET2 | p.R1216X | 30.5 |
| P146 | ZRSR2 | p.R290X | 35.7 |
| P146 | TET2 | p.S765Kfs*4 | 19.5 |
| P147 | DNMT3A | p.R736C | 6.3 |
| P148 | TET2 | p.E1320Vfs*15 | 6.3 |
| P148 | DNMT3A | p.I310T | 9.1 |
| P148 | TET2 | p.Q591* | 7.3 |
| P149 | TET2 | p.A912fs | 13.2 |
| P149 | SF3B1 | p.K666N | 6.6 |
| P150 | U2AF1 | p.S34F | 2.8 |
| P151 | ASXL1 | p.S1027Gfs*4 | 2.6 |
| P151 | NF1 | p.I679Dfs*21 | 9.8 |
| P151 | SRSF2 | p.P95_R102del | 33.6 |
| P152 | CBL | p.C416S | 14.2 |
| P153 | NRAS | p.G12S | 5.4 |
| P154 | KRAS | p.G12D | 23.4 |
| P155 | DNMT3A | p.V341fs | 32.2 |
| P156 | PRF1 | p.P22Rfs*29 | 2.6 |
| P157 | KMT2D | p.R625Afs*305 | 3.3 |
| P158 | DNMT3A | p.R882C | 38.4 |
| P159 | CD79B | p.V222Cfs*3 | 3.5 |
| P160 | DNMT3A | p.R882C | 2.3 |
| P161 | KRAS | p.G12D | 40.0 |
| P162 | PTPN11 | p.D61V | 9.0 |
| P163 | KRAS | p.A146T | 14.6 |
| P164 | TET2 | p.N1765Kfs*24 | 32.6 |
| P165 | DNMT3A | p.R882H | 8.1 |
| P166 | DNMT3A | p.E371Gfs*36 | 19.8 |
| P167 | PPM1D | p.E540X | 34.1 |
| P168 | DNMT3A | p.R688H | 2.2 |
| P169 | ZRSR2 | p.D432_R435del | 33.2 |
| P170 | KMT2D | p.L1603fs*1 | 3.8 |
| P171 | U2AF1 | p.S34F | 20.8 |
| P172 | DNMT3A | p.F732del | 28.0 |
| P173 | DNMT3A | p.R882H | 26.0 |
| P174 | U2AF1 | p.S34Y | 34.7 |
| P175 | RBBP6 | p.N1134Mfs*23 | 14.7 |
| P175 | DNMT3A | p.Q573X | 13.1 |
| P176 | DDX41 | p.G530D | 20.6 |
| P176 | SF3B1 | p.K666N | 5.8 |
| P177 | ASXL1 | p.N703Mfs*22 | 20.0 |
| P177 | BRAF | p.G596R | 15.0 |
| P178 | PPM1D | p.L490Ffs*3 | 4.6 |
| P179 | SPEN | p.S2306del | 12.5 |
| P179 | CBL | p.R506X | 3.3 |
| P180 | KMT2D | p.R2915X | 7.3 |
| P181 | DNMT3A | p.F732delF | 11.9 |
| P182 | DNMT3A | p.R458Gfs*193 | 2.0 |
| P183 | DNMT3A | p.M880fs*1 | 3.3 |
| P184 | STAT3 | p.Y640F | 3.1 |
| P185 | DNMT3A | p.C494Lfs*52 | 4.1 |
| P186 | CBL | p.R420Q | 3.0 |
| P187 | DNMT3A | p.E774X | 6.7 |
| P188 | U2AF1 | p.S34F | 33.7 |
| P189 | TRAF3 | p.K286Efs*11 | 7.9 |
| P190 | PPM1D | p.R572* | 10.1 |
| P190 | ZRSR2 | p.Q213* | 8.4 |
| P191 | STAT3 | p.E616G | 2.1 |
| P192 | SETD1B | p.Q1589Sfs*11 | 2.1 |
| P192 | DNMT3A | p.S770* | 26.8 |
| P193 | DNMT3A | p.E561Afs*17 | 6.3 |
| P194 | BCOR | p.E1093X | 4.0 |
| P195 | CBL | p.R420Q | 6.2 |
| P196 | ASXL1 | p.Y591* | 20.5 |

Pt: Patient; AA: Amino acids；VAF: Variant allele frequency.

**Table S4. Baseline characteristics of all patients in cohort 2.**

| **Characteristics** | **ITP-non-CH**  **(n=492)** | **ITP-CH**  **(n=47)** | ***P* - value** |
| --- | --- | --- | --- |
| Age, year (median, IQR) | 45 (28.3, 57) | 59 (46, 68) | <0.001 |
| Sex, n (%)  Female  Male | 302 (61.4)  190 (38.6) | 20 (42.6)  27 (57.4) | 0.012 |
| WBC, ×10^9^/L (median, IQR) | 6.6 (5.1, 9.2) | 5.9 (4.6, 8.3) | 0.112 |
| RBC, ×10^12^/L (median, IQR) | 4.5 (4.1, 5.0) | 4.2 (3.7, 4.6) | 0.002 |
| Hb, g/L (median, IQR) | 131 (117.3, 146.8) | 134 (123, 142) | 0.643 |
| PLT, ×10^9^/L (median, IQR) | 19.5 (7, 35) | 28 (19, 47) | 0.007 |
| ANC, ×10^9^/L (median, IQR) | 4.2 (2.9, 6.2) | 3.6 (2.5, 5.4) | 0.056 |
| Neutrophils percentage, % (median, IQR) | 63.8 (54.5, 72.6) | 59.1 (52.6, 69.4) | 0.096 |
| LDH, U/L (median, IQR) | 206.8 (176, 255.4) | 207.5(175, 255) | 0.802 |
| Most common previous treatment, no.(%)  Glucocorticoids | n=310  217 (70) | n=15  10 (66.7) | 0.907 |
| rhTPO | 143 (46.1) | 4 (26.7) | 0.332 |
| IVIg | 123 (39.7) | 2 (13.3) | 0.083 |
| TPO-RAs | 74 (23.9) | 1 (6.7) | 0.191 |
| Cyclosporine | 36 (11.6) | 1 (6.7) | 0.592 |
| Danazol | 29 (9.4) | 0 (0) | 0.237 |

IQR: interquartile range; ITP-non-CH: immune thrombocytopenia (ITP) without gene mutation associated with clonal hematopoiesis (CH). WBC: white blood cell count; RBC: red blood cell count; ANC: absolute neutrophil count; Hb: hemoglobin; PLT: platelet count; ANC: absolute neutrophil count; LDH: lactate dehydrogenase; IVIg: intravenous immunoglobulin; rhTPO: recombinant human thrombopoietin (TPO); TPO-RAs: TPO receptor agonists.

**Table S5. Details of gene mutation profiles associated with clonal hematopoiesis (CH) in ITP patients in cohort 2.**

| **Pt** | **Gene** | **AA_change** | **VAF (%)** |
| --- | --- | --- | --- |
| P1 | TET2 | p.C973* | 8.7 |
| P1 | TET2 | p.E1162Rfs*8 | 6.1 |
| P1 | PPM1D | p.R552* | 23.1 |
| P2 | BCOR | p.I1511Mfs*23 | 16.7 |
| P3 | U2AF2 | p.K87del | 3 |
| P4 | ZRSR2 | p.S447_R448del | 18.2 |
| P4 | DNMT3A | p.W327* | 2.1 |
| P5 | DNMT3A | p.Q692* | 2.1 |
| P5 | TET2 | p.I1873T | 2 |
| P6 | DNMT3A | p.R771* | 2.3 |
| P7 | TET2 | p.L1322P | 2.4 |
| P8 | U2AF1 | p.S34Y | 3.4 |
| P9 | DNMT3A | p.R882H | 2.7 |
| P10 | DNMT3A | p.I310F | 14.4 |
| P11 | BCOR | p.Q1274* | 4.6 |
| P12 | ASXL1 | p.G646Wfs*12 | 2.5 |
| P13 | DNMT3A | p.K826R | 3.7 |
| P14 | DNMT3A | p.K766Efs*15 | 14 |
| P15 | ASXL1 | p.Y700* | 2.6 |
| P16 | ZRSR2 | p.R452_R454dup | 2.7 |
| P17 | TET2 | p.R1516* | 2.3 |
| P18 | ETV6 | p.E76* | 2.8 |
| P19 | DNMT3A | p.R882C | 4.8 |
| P20 | BCOR | p.R1513* | 7.2 |
| P21 | KMT2C | p.Q4514* | 5.8 |
| P22 | DNMT3A | p.C559* | 2.5 |
| P23 | DNMT3A | p.R803K | 3.8 |
| P24 | NF1 | p.L581Ffs*6 | 17.1 |
| P25 | DNMT3A | p.Y683Vfs*30 | 2.3 |
| P26 | TET2 | p.W1291* | 6.1 |
| P27 | BCOR | p.I1423Lfs*27 | 3.1 |
| P28 | TET2 | p.L1326* | 13.5 |
| P29 | TET2 | p.R1516* | 11.8 |
| P30 | DNMT3A | p.Q615* | 3.3 |
| P31 | ASXL1 | p.R404* | 2.1 |
| P32 | TET2 | p.S602Ffs*3 | 6.8 |
| P33 | DNMT3A | p.R320* | 5 |
| P34 | DNMT3A | p.W297* | 10.5 |
| P35 | IDH2 | p.S227Gfs*52 | 15 |
| P36 | TET2 | p.E1250* | 3.5 |
| P37 | DNMT3A | p.S770Lfs*12 | 2.1 |
| P38 | U2AF1 | p.S34Y | 11.5 |
| P39 | DNMT3A | p.R771X | 7.2 |
| P40 | DNMT3A | p.R736C | 11 |
| P41 | DNMT3A | p.E733* | 4.7 |
| P42 | DNMT3A | p.E907Sfs*15 | 3.1 |
| P43 | BCOR | p.T437Nfs*3 | 3.83 |
| P44 | PPM1D | p.R552* | 3.08 |
| P45 | DNMT3A | p.E725* | 4.54 |
| P46 | DNMT3A | p.R882H | 2.17 |
| P47 | DNMT3A | p.L723Pfs*56 | 2.32 |

Pt: Patient; AA: Amino acids；VAF: Variant allele frequency.

**Table S6. OPLS-DA model validation metrics for all group comparisons.**

| Index | ITP-non-CH vs. ITP-CH | Epigenetic vs. RNA Splicing vs. Transcription | DNMT3A R882 vs. non-R882 |
| --- | --- | --- | --- |
| R²Y | 0.0493 | 0.3490 | 0.6480 |
| Q² | 0.0405 | 0.1810 | 0.4830 |
| CV-ANOVA P | 1.542e-05 | 1.51e-02 | 1.364e-03 |

**Table S7. Differential metabolic between patients with *DNMT3A*-R882 and *DNMT3A*-non-R882 mutated clonal hematopoiesis.**

| **Compound** | **VIP** | **Fold_Change** | **Log2FC** | **P-value** |
| --- | --- | --- | --- | --- |
| Choline | 1.669 | 0.806 | -0.311 | 0.001 |
| Biliverdin | 2.551 | 0.356 | -1.489 | 0.005 |
| 2,3-Dihydrobenzofuran | 1.349 | 0.621 | -0.687 | 0.006 |
| Bilirubin (E-E) | 2.141 | 0.188 | -2.411 | 0.007 |
| Hydroxyacetone | 1.460 | 0.483 | -1.049 | 0.032 |
| Dimethylglycine | 1.329 | 0.665 | -0.589 | 0.002 |
| Glycochenodeoxycholic acid | 1.838 | 0.177 | -2.499 | 0.005 |
| 8,9-Epoxyeicosatrienoic acid | 1.194 | 1.438 | 0.524 | 0.002 |
| 12-Ketolithocholic acid | 1.139 | 0.235 | -2.089 | 0.041 |
| Glu-Ile | 1.871 | 0.255 | -1.969 | 0.008 |
| 2-Phenylacetamide | 1.770 | 0.691 | -0.534 | 0.002 |
| Isocorydine | 1.466 | 0.493 | -1.021 | 0.008 |
| Isopentenyl pyrophosphate | 1.762 | 0.699 | -0.518 | 0.003 |
| Gly-Val | 1.438 | 1.217 | 0.284 | 0.011 |
| Val-Gln | 2.189 | 1.584 | 0.664 | 0.000 |
| Iodotyrosine | 1.820 | 0.464 | -1.108 | 0.015 |
| Dodecanoic acid | 1.754 | 1.491 | 0.577 | 0.011 |
| 1-Palmitoyl-2-oleoyl-sn-glycero-3-phosphocholine | 1.185 | 1.254 | 0.326 | 0.005 |
| Phytosphingosine | 1.618 | 1.276 | 0.351 | 0.003 |
| L-glyceraldehyde | 1.450 | 0.539 | -0.892 | 0.013 |
| 4-Hydroxyphenylglyoxylic acid | 1.703 | 1.212 | 0.278 | 0.011 |
| Citramalic acid | 1.271 | 0.501 | -0.998 | 0.041 |
| Dimethylarsinic acid | 1.826 | 0.670 | -0.578 | 0.030 |
| 5,8,11-Eicosatrienoic acid | 1.161 | 1.363 | 0.447 | 0.042 |
| m-Chlorohippuric acid | 1.832 | 0.541 | -0.888 | 0.000 |
| Tyr-Ile | 2.289 | 0.399 | -1.327 | 0.024 |
| Diethyl glutarate | 1.766 | 1.386 | 0.470 | 0.022 |
| Aconitic acid | 2.489 | 0.298 | -1.745 | 0.000 |
| Ethisterone | 1.728 | 0.564 | -0.827 | 0.005 |
| 2-Aminoadenosine | 1.549 | 0.514 | -0.961 | 0.003 |
| Carnitine C14:2 | 1.260 | 0.510 | -0.972 | 0.007 |
| Spermidic acid | 1.893 | 0.707 | -0.501 | 0.000 |
| PE(20:2(11Z,14Z)/18:2(9Z,12Z)) | 2.009 | 1.384 | 0.469 | 0.016 |
| 1-Methylpyrrolidine-2-carboxylic acid | 1.514 | 1.463 | 0.549 | 0.014 |
| 2-[(Pyrrolidinium-2-ylcarbonyl)amino]propanoate | 1.020 | 0.459 | -1.123 | 0.043 |
| 1,2-Dimyristoyl-sn-glycero-3-phospho-l-serine | 2.579 | 0.363 | -1.460 | 0.042 |
| Glycocholic acid | 1.106 | 0.198 | -2.340 | 0.006 |
| (±)13-HpODE | 1.455 | 0.627 | -0.673 | 0.006 |
| α-Ketoglutaric Acid (α-KG) | 1.717 | 0.476 | -1.072 | 0.016 |
| Succinic acid | 1.034 | 0.789 | -0.342 | 0.038 |
| Sebacic acid | 1.454 | 0.698 | -0.519 | 0.015 |
| Bilirubin | 2.483 | 0.189 | -2.404 | 0.014 |
| Mannitol | 1.076 | 0.717 | -0.481 | 0.043 |
| Deoxycholic acid glycine conjugate | 1.542 | 0.150 | -2.737 | 0.005 |
| Ursodeoxycholic acid | 1.238 | 0.561 | -0.835 | 0.001 |
| Xylose | 1.343 | 1.385 | 0.470 | 0.002 |
| N-Methyltryptamine | 1.439 | 0.204 | -2.291 | 0.041 |
| 5,15-DiHETE | 2.010 | 0.503 | -0.990 | 0.015 |
| Glyceraldehyde | 1.862 | 0.660 | -0.599 | 0.009 |
| 8,15-Dihete | 1.992 | 0.565 | -0.824 | 0.016 |
| Allopurinol | 1.489 | 0.462 | -1.114 | 0.012 |
| 13(S)-Hydroperoxylinolenic acid | 1.082 | 0.675 | -0.567 | 0.029 |
| Dihomo-gamma-linolenic acid | 1.269 | 0.572 | -0.807 | 0.015 |
| 9-Octadecynoic acid | 1.392 | 0.640 | -0.643 | 0.049 |
| alpha-Linolenic acid | 1.649 | 0.696 | -0.524 | 0.014 |
| 5,8,14-Eicosatrienoic acid, 11,12-dihydroxy-, (5Z,8Z,14Z)- | 1.508 | 0.587 | -0.769 | 0.022 |
| h-gamma-Glu-leu-oh | 1.953 | 0.269 | -1.896 | 0.008 |

**Table S8. Differential metabolic between ITP patients with and without gene mutation associated with clonal hematopoiesis.**

| **Compound** | **VIP** | **Fold_Change** | **Log2FC** | ***P* value** |
| --- | --- | --- | --- | --- |
| Biliverdin | 2.816 | 0.771 | -0.375 | 0.034 |
| 6-Benzylaminopurine | 1.731 | 1.364 | 0.448 | 0.025 |
| Tyr-Ile | 1.110 | 1.362 | 0.445 | 0.027 |
| Carnitine C14:2 | 4.058 | 0.677 | -0.563 | 0.011 |
| 1-(9Z-heptadecenoyl)-sn-glycero-3-phosphoethanolamine | 1.264 | 1.260 | 0.334 | 0.020 |
| LPE(16:1/0:0) | 1.935 | 1.295 | 0.373 | 0.025 |
| Glycocholic acid | 1.203 | 1.448 | 0.534 | 0.042 |
| Undecanedioic acid | 1.647 | 1.345 | 0.427 | 0.000 |
| prostaglandin B2 | 1.298 | 1.523 | 0.607 | 0.000 |
| Isodeoxycholic acid | 2.070 | 1.320 | 0.401 | 0.008 |
| 3,4-Dihydroxymandelic acid | 1.182 | 1.443 | 0.529 | 0.011 |
| N-Acetyl-D-glucosamine | 1.136 | 1.215 | 0.281 | 0.006 |
| prostaglandin E3 | 2.081 | 0.793 | -0.335 | 0.036 |
| LPE(20:3/0:0) | 2.163 | 1.327 | 0.409 | 0.000 |

**Table S9. Multi-model performance comparison: training and test sets (all configurations).**

| **Model** | **Feature Set** | **Accuracy** | **F1** | **AUC** | **TP** | **TN** | **FP** | **FN** |
| --- | --- | --- | --- | --- | --- | --- | --- | --- |
| Train |  |  |  |  |  |  |  |  |
| LR | 14 Met | 0.674 | 0.281 | 0.762 | 24 | 230 | 114 | 9 |
| **LR** | **Met+3Clin ★** | **0.735** | **0.342** | **0.841** | **26** | **251** | **93** | **7** |
| LR | Met+5Clin | 0.751 | 0.356 | 0.856 | 26 | 257 | 87 | 7 |
| MLP | 14 Met | 0.920 | 0.250 | 0.639 | 5 | 342 | 2 | 28 |
| MLP | Met+3Clin | 0.928 | 0.372 | 0.543 | 8 | 342 | 2 | 25 |
| MLP | Met+5Clin | 0.912 | 0.000 | 0.666 | 0 | 344 | 0 | 33 |
| RF | Met+3Clin | 0.992 | 0.957 | 0.999 | 33 | 341 | 3 | 0 |
| GBM | Met+3Clin | 1.000 | 1.000 | 1.000 | 33 | 344 | 0 | 0 |
| SVM | Met+3Clin | 0.727 | 0.376 | 0.922 | 31 | 243 | 101 | 2 |
| LDA | Met+3Clin | 0.918 | 0.311 | 0.837 | 7 | 339 | 5 | 26 |
| KNN | Met+3Clin | 1.000 | 1.000 | 1.000 | 33 | 344 | 0 | 0 |
| Ensemble | Met+3Clin | 0.989 | 0.935 | 1.000 | 29 | 344 | 0 | 4 |
| Test |  |  |  |  |  |  |  |  |
| LR | 14 Met | 0.654 | 0.152 | 0.599 | 5 | 101 | 47 | 9 |
| **LR** | **Met+3Clin ★** | **0.735** | **0.218** | **0.721** | **6** | **113** | **35** | **8** |
| LR | Met+5Clin | 0.735 | 0.189 | 0.698 | 5 | 114 | 34 | 9 |
| MLP | 14 Met | 0.914 | 0.125 | 0.553 | 1 | 147 | 1 | 13 |
| MLP | Met+3Clin | 0.901 | 0.111 | 0.388 | 1 | 145 | 3 | 13 |
| MLP | Met+5Clin | 0.914 | 0.000 | 0.688 | 0 | 148 | 0 | 14 |
| RF | Met+3Clin | 0.870 | 0.000 | 0.619 | 0 | 141 | 7 | 14 |
| GBM | Met+3Clin | 0.907 | 0.118 | 0.634 | 1 | 146 | 2 | 13 |
| SVM | Met+3Clin | 0.704 | 0.143 | 0.666 | 4 | 110 | 38 | 10 |
| LDA | Met+3Clin | 0.883 | 0.174 | 0.734 | 2 | 141 | 7 | 12 |
| KNN | Met+3Clin | 0.914 | 0.000 | 0.709 | 0 | 148 | 0 | 14 |
| Ensemble | Met+3Clin | 0.895 | 0.105 | 0.682 | 1 | 144 | 4 | 13 |

Values shown are from logistic regression (LR), multilayer perceptron (MLP), random forest (RF), gradient boosting (GBM), support vector machine (SVM), linear discriminant analysis (LDA), k-nearest neighbors (KNN), and soft-voting ensemble. All confusion matrices verified: TP + TN + FP + FN = N for all entries.

Note: MLP results are from a representative single training run (random_state = 42); due to stochastic weight initialization, MLP AUC values may vary across runs.

**Table S10. Complete parameter table of the logistic regression model (14 metabolites + 3 clinical features: sex, age, PLT).**

| **#** | **Feature** | **β (Coefficient)** | **μ (Train Mean)** | **σ (Train SD)** |
| --- | --- | --- | --- | --- |
| 1 | Biliverdin | +0.081714 | 50974901.8161 | 42044342.3656 |
| 2 | 6-Benzylaminopurine | -0.074547 | 9470870.6877 | 9482099.2161 |
| 3 | Tyr-Ile | -0.080622 | 15787227.9917 | 37621640.3352 |
| 4 | Carnitine C14:2 | +0.214340 | 88324562.5900 | 63125591.7593 |
| 5 | 1-(9Z-heptadecenoyl)-sn-glycero-3-phosphoethanolamine | -0.106251 | 4923950.3021 | 4913824.5646 |
| 6 | LPE(16:1/0:0) | -0.027528 | 21909887.5783 | 21059167.9135 |
| 7 | Glycocholic acid | -0.141796 | 103589643.1677 | 184484397.3188 |
| 8 | Undecanedioic acid | -0.161223 | 61532886.1281 | 42719112.0546 |
| 9 | prostaglandin B2 | -0.081293 | 4294180.7307 | 5785535.6824 |
| 10 | Isodeoxycholic acid | -0.073391 | 6100363.2593 | 5825615.9708 |
| 11 | 3,4-Dihydroxymandelic acid | -0.117481 | 52337714.5436 | 71386976.1321 |
| 12 | N-Acetyl-D-glucosamine | -0.142892 | 7761095.9812 | 5958118.5653 |
| 13 | prostaglandin E3 | +0.052712 | 31441604.8724 | 21604694.5121 |
| 14 | LPE(20:3/0:0) | -0.163731 | 42827127.2386 | 24419248.8640 |
| 15 | Sex | -0.126798 | 0.5729 | 0.4947 |
| 16 | age | +0.350613 | 43.9045 | 18.1426 |
| 17 | PLT | +0.117151 | 26.0424 | 22.0138 |

**Table S11. List of 141-genes panel.**

| **Genes** | **Genes** | **Genes** | **Genes** | **Genes** | **Genes** | **Genes** |
| --- | --- | --- | --- | --- | --- | --- |
| ABL1 | CCR4 | NOTCH3 | KDM6A | PIGA | SPEN | ZRSR2 |
| ANKRD26 | CD28 | EP300 | KIT | NOTCH4 | SRP72 | PRPF8 |
| ARID1A | CD58 | ETNK1 | KLF2 | PLCG1 | SRSF2 | ZNF384 |
| ASXL1 | CD79B | ETV6 | KMT2A | PLCG2 | STAG2 | CCND3 |
| ASXL2 | CDC25C | EZH2 | KMT2D | PPM1D | STAT3 | EGR1 |
| ATG2B | CDKN1B | FAM46C | KRAS | PRDM1 | STAT5B | JAK3 |
| ATM | CDKN2A | FAT1 | MAP2K1 | PRKCB | SUZ12 | PHF6 |
| B2M | CEBPA | FBXW7 | MAPK1 | PRPS1 | TAL1 | SMC3 |
| BCL2 | CNOT3 | FGFR3 | MAX | PTEN | TCF3 | ZMYM3 |
| BCL6 | CREBBP | FLT3 | MED12 | PTPN11 | TERT |  |
| BCOR | CRLF2 | GATA1 | MEF2B | RAD21 | TET2 |  |
| BCORL1 | CSF3R | GATA2 | MPL | RBBP6 | TNFAIP3 |  |
| BIRC3 | CSNK1A1 | GATA3 | MYC | RELN | TNFRSF14 |  |
| BRAF | CUX1 | GNA13 | MYD88 | RHOA | TP53 |  |
| BRINP3 | CXCR4 | ID3 | NF1 | RPL10 | TPMT |  |
| BTK | DDX3X | IDH1 | NOTCH1 | RUNX1 | TRAF3 |  |
| CALR | DDX41 | IDH2 | NOTCH2 | SETBP1 | U2AF1 |  |
| CARD11 | DIS3 | IKZF1 | NPM1 | SETD2 | USP7 |  |
| CASP8 | DNM2 | IL7R | NRAS | SF1 | WHSC1 |  |
| CBL | DNMT3A | IRF4 | NT5C2 | SF3B1 | WT1 |  |
| CCND1 | DNMT3B | JAK1 | PAX5 | SH2B3 | XPO1 |  |
| CCND2 | EED | JAK2 | PDGFRB | SMC1A | ZBTB7A |  |

**Table S12. List of 267-genes panel.**

| **Genes** | **Genes** | **Genes** | **Genes** | **Genes** | **Genes** | **Genes** |
| --- | --- | --- | --- | --- | --- | --- |
| ABCB1 | ABL1 | ANKRD26 | APC | ARID1A | ARID1B | ARID2 |
| ATG2B | ATM | ATRX | B2M | BACH2 | BCL10 | BCL2 |
| BCORL1 | BIRC3 | BLM | BPGM | BRAF | BRCA1 | BRCA2 |
| BTK | CALR | CARD11 | CBL | CBLB | CBLC | CCND1 |
| CD58 | CD79A | CD79B | CDC25C | CDKN1A | CDKN1B | CDKN2A |
| CHD2 | CHD8 | CIITA | CNOT3 | CREBBP | CRLF2 | CSF1R |
| CTCF | CUX1 | CXCR4 | CYLD | DDX3X | DDX41 | DIS3 |
| DNMT3B | DTX1 | DUSP2 | EBF1 | EED | EGFR | EGLN1 |
| EPHA7 | EPOR | ETNK1 | ETV6 | EZH2 | FAM46C | FAS |
| FBXW7 | FGFR3 | FLT3 | FOXO1 | FYN | GAB2 | GATA1 |
| GNA13 | GNAI2 | GNAS | GNB1 | GSKIP | H1-2 | H1-3 |
| HLA-A | HLA-C | HLA-DMB | HNRNPK | HRAS | HUWE1 | HVCN1 |
| IGLL5 | IKZF1 | IKZF2 | IKZF3 | IL7R | IRF2BP2 | IRF4 |
| JAK2 | JAK3 | JUNB | KDM6A | KIT | KLF2 | KLHL6 |
| KMT2D | KRAS | KRT20 | LCOR | LMO2 | LTB | LYN |
| MCL1 | MED12 | MEF2B | MFHAS1 | MPL | MTOR | MYC |
| NF1 | NFE2 | NFKBIA | NFKBIE | NOTCH1 | NOTCH2 | NOTCH3 |
| NT5C2 | P2RY8 | PALB2 | PAX5 | PDGFRA | PDGFRB | PDS5B |
| PIK3CD | PIK3R1 | PIM1 | PIM2 | PLCG1 | PLCG2 | POT1 |
| PRKCB | PRKD2 | PRKDC | PRPF8 | PRPS1 | PSMB5 | PTEN |
| RAD21 | RASA2 | RB1 | RBBP6 | RELN | RHOA | RPL10 |
| SBDS | SETBP1 | SETD1B | SETD2 | SETDB1 | SF1 | SF3B1 |
| SMARCA4 | SMC1A | SMC3 | SMO | SOCS1 | SP140 | SPEN |
| STAT3 | STAT5B | STAT6 | SUFU | SUZ12 | SYK | TAL1 |
| TERT | TET1 | TET2 | TMEM30A | TMSB4X | TNFAIP3 | TNFRSF14 |
| TRAF3 | U2AF1 | UBE2A | UBR5 | USP7 | VAV1 | VHL |
| XPO1 | ZAP70 | ZBTB7A | ZFP36L1 | ZMYM3 | ZNF292 | ZRSR2 |
| ARID5B | ASXL1 | ASXL2 | BCL6 | BCL7A | BCOR | BRIP1 |
| BTG1 | BTG2 | CCND3 | CCR4 | CD28 | CDKN2B | CDKN2C |
| CEBPA | CSF3R | CSMD1 | CSNK1A1 | DKC1 | DNM2 | DNMT3A |
| EGR1 | ELANE | EP300 | FAT1 | FAT4 | FBXO11 | GATA2 |
| GATA3 | GFI1 | H1-4 | H1-5 | HAX1 | ID3 | IDH1 |
| IDH2 | IRF8 | ITPKB | JAK1 | KMT2A | KMT2B | KMT2C |
| MAP2K1 | MAPK1 | MAX | MYCN | MYD88 | MYOM2 | NOTCH4 |
| NPM1 | NRAS | PHF6 | PIGA | PIK3CA | PPM1D | PRDM1 |
| PRF1 | PTPN1 | PTPN11 | PTPRD | RRAGC | RUNX1 | SAMHD1 |
| SGK1 | SH2B3 | SH2D1A | SRP72 | SRSF2 | STAG2 | TBL1XR1 |
| TCF3 | TERC | TOX | TP53 | TPMT | WHSC1 | WT1 |
| XBP1 |  |  |  |  |  |  |
